# New Teeth

**Published:** 1865

**Authors:** 


### NEW TEETH.

We have recently been using teeth manufactured by Duff & Co., which for natural life like appearance we have scarcely seen excelled. There are amongst them some very desirable forms. We must say, however, that, including all the teeth manufactured, there is quite a deficiency in regard to form; in this fact is an argument for a greater number of manufacturers. We have little or no criticism to make upon most of the teeth that are made, but that a greater variety in size, shape and shade is required none will deny, but improvement is all the time being made. Every manufacturer has some excellencies peculiar to himself.

